# Antrochoanal polyp: a review of sixteen cases

**DOI:** 10.1016/S1808-8694(15)31052-1

**Published:** 2015-10-19

**Authors:** Marcos Rabelo de Freitas, Rogério Pinto Giesta, Sebastião Diógenes Pinheiro, Viviane Carvalho da Silva

**Affiliations:** 1 M.S. in Otorhinolaryngology - Medical School of Ribeirão Preto/USP, Assistant Professor and Head of the Medical Residence in Otorhinolaryngology - Walter Cantídio University Hospital and the Medical School of the Federal University of Ceará.; 2 M.S. Student at the Pathology and Forensic Medicine Department of the Medical School of the Federal University of Ceará; Substitute Professor - Pathology Department - Medical School - Federal University of Ceará.; 3 PhD. Associate Professor and Head of the Otorhinolaryngology Department - Walter Cantídio University Hospital and Medical School of the Federal University of Ceará.; 4 M.S. Student - Department of Community Health - Medical School of the Federal University of Ceará; Assistant Physician - Department of Otorhinolaryngology - Walter Cantídio University Hospital and Medical School of the Federal University of Ceará.

**Keywords:** antrochoanal polyp, killian’s polyp, surgical treatment

## Abstract

Introduction: An Antrochoanal polyp, or Killian’s polyp is a benign solitary polypoid lesion that affects mainly children and young adults. Studies demonstrate that Killin’s polyp generally represents 4-6% of all nasal polyps. However, in the pediatric population this percentage reaches 33%. It originates from a hypertrophy of the mucous membrane on the maxillary sinus antrum, and it grows for unknown reasons, through the maxillary sinus ostium towards the nasal cavity and the choana - the posterior portion of the pharynx. **Aim:** To evaluate the result of the surgical treatment on patients assisted in the Department of Otorhinolaryngology of the Walter Cantídio University Hospital - Medical School of the Federal University of Ceará, mainly on the surgical technique employed and the efficacy of each technique in controlling the disease. **Materials and methods:** Retrospective study, accomplished through a chart analysis from the patients submitted to polypectomy because of Killian’s polyps or other nasal polyps, operated from March 1st, 1991 to April 30th of 2001, in the Department of Otorhinolaryngology of the Medical School of the Walter Cantídio University Hospital of the Federal University of Ceará. **Results:** Nine patient (56.6%) were males and 7 (43.8%) were females. Eleven (68.75%) patients were between 8 and 20 years of age. Predominant symptoms were unilateral nasal obstruction (81.3%) and purulent rhinorrhoea (43.8%). The most common procedure employed was the combined approach: external and endonasal, in 87.5% of the cases. Antrochoanal polyp removal procedure accounted for 21.6% of all the surgical procedures accomplished in the same period for the removal of nasal polyps. Postoperative recurrence was of 12.5%. **Conclusions:** Antrochoanal polyp was an affection that prevailed among children and young adults. The combined external and endonasal approach was the one most used. Despite maxillary sinus approach to the polyp origin, postoperative recurrence is a possibility.

## INTRODUCTION

The antrochoanal polyp, or Killian’s Polyp is a benign, solitary lesion that affect mainly children and young adults. Studies show that Killian’s Polyp accounts for 4-6% of all nasal polyps that affect the general population; however, in the pediatric population this percentage reaches 33% ^1^. It sprouts from a hypertrophy of the maxillary sinus mucosa, near its ostium, and develops because of some unknown stimuli, through the maxillary sinus ostium, towards the nasal cavity and the choana, growing all the way to the nasopharynx; it may even reach the oropharynx in some instances^11^. It rarely comes from other regions, such as the sphenoid or ethmoid sinuses^3^. Polyps grow quickly, probably due to the venous return of is pedicle, which is then compressed by the ostium opening, thus making the polyp increasingly more edematous. Its clinical manifestation is, usually, unilateral nasal obstruction; however, it may become bilateral in those cases in which the polyp is extremely bulky, with relevant nasal septum deviation. It is followed by mucous or mucous-purulent nasal secretion; and it may eventually obstruct the Eustachian tube ostium, thus causing secretory otitis media. Epistaxis may be a non-classical sign of a Killian’s polyp^10^. We notice that the maxillary sinus ostium in enlarged in its diameter because of the polyp pedicle, which would then increase its dimensions. Histopathology shows Killian’s polyps as a central cystic cavity, surrounded by edema and with an external wall coated by normal respiratory tissue. In some cases, we find microcysts inside them; however no glandular structures can be found.

Its first description was made in 1753, by Palfyn, when he reported on a woman with a nasopharynx polyp growing towards her uvula. Notwithstanding, it was only in 1906 when Killian, for the first time, provided a precise description of the antrochoanal polyp natural history, stating that its origin was in the maxillary sinus, and not in the posterior choana, as it was previously believed. Notwithstanding, Killian was unable to confirm its origin in the maxillary antrum, which was then done by Kubo in 1909^11^. On physical exam, by means of an anterior rhinoscopy or a nasofibroscope, it is possible to see a single polyp leaving the middle meatus and projecting itself in the choana, partially or totally filling the cavum. On CT scan we see a homogeneous filling and turning opaque the maxillary sinus cavity, of the ipsilateral nasal cavity and, eventually, involving the cavum, we may also see a nasal wall deviation, specially that of the nasal septum; however, without bone destruction. This is considered the best image exam for this disease. CT scans also help in choosing the surgical technique to be used, since it shows clearly all the tumor mass boundaries. The major differential diagnosis is made with retention mucous cysts, maxillary antrum tumor, mucocele, maxillary sinusitis and meningocelephalocele. Treatment is surgery-based, and there are many access approaches to the polyp. The ones most used are the endonasal microsurgery, endoscopic surgery and the Caldwell-Luc4 approach. Despite following rigorously the advocated surgical techniques, the polyp still recurs with relative frequency. That is why, most of the times, it is recommended to explore the antrum and remove the maxillary sinus disease.

## OBJECTIVE

To describe sixteen cases of patients with a histopathologic diagnosis of antrochoanal polyp, seen at the Department of Otorhinolaryngology of the Walter Cantídio University Hospital of the Medical School of the Federal University of Ceará.

## MATERIALS AND METHODS

This is a longitudinal historic cohort carried out through the analysis of Killian or other polyp type patients’ charts, who underwent polypectomy during March 1st, 1991 through April 30th of 2001, at the Otolaryngology Department of the Walter Cantídio University Hospital - Federal University of Ceará. This study was approved by the Ethics in Research Committee of this Medical School.

## RESULTS

**Chart 1:**
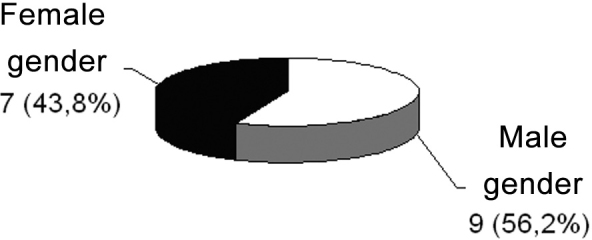
Gender relation

**Chart 2:**
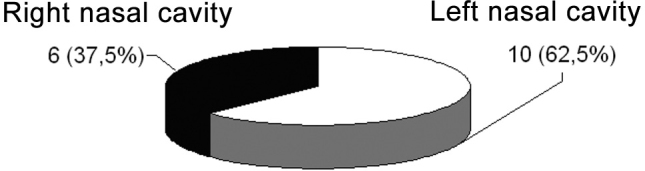
Polyp location

After reviewing these sixteen cases, we had nine male patients (56.2%) and seven females (43.8%) (Chart 1), and in ten of these patients (62.5%), the polyp was located in the left nasal cavity and, in six (37.5%), it was in the right side nasal cavity (Chart 2).

**Chart 3:**
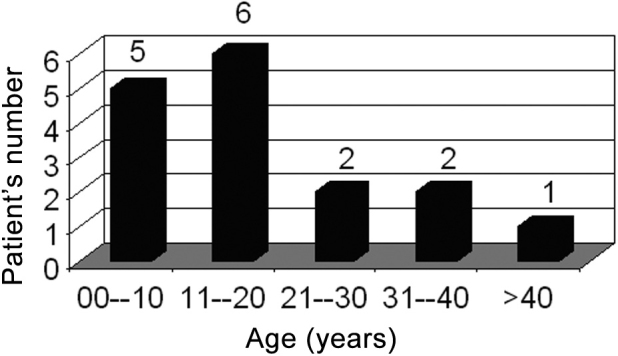
Patient’s age

Age ranged between 8 and 43 years, and most of the cases were between 8 and 20 years (Chart 3).

The major symptoms seen were constant and unilateral nasal obstruction in thirteen patients (81.3%), purulent rhinorrhea in seven (43.8%), epistaxis in six (37.5%), clear rhinorrhea in four (25%), snoring and facial pain in three patients (18.8%); and also bilateral nasal obstruction, dysphagia, oral bleeding, halitosis, sniffing and head ache in two patients; and dysphonia, dyspnea, nasal pruritus, anosmia and cacosmia in one patient (Chart 1).

**Chart 4:**
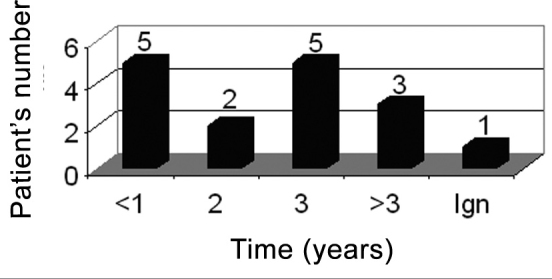
Time of symptoms until specialized care

Average time between symptoms onset and the medical visit was of three years (Chart 4).

**Chart 5:**
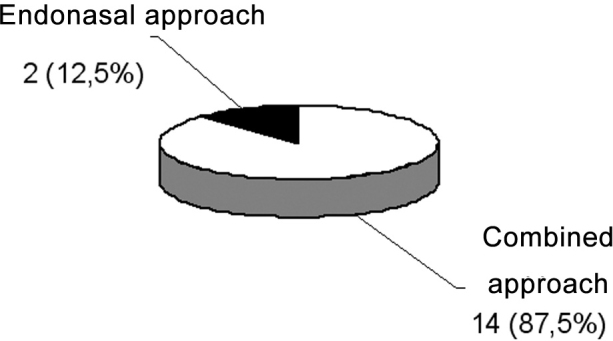
Surgeries

As to the surgical approaches used, two patients (12.5%) underwent polyp removal by endonasal access and the others underwent an endonasal and Caldwell-Luc combined approach (Chart 5).

Of these, sixteen patients had already undergone anterior polypectomy, one of them had suffered it one year and a half in this same institution, and the other had it five years before, in another medical facility.

**Chart 6:**
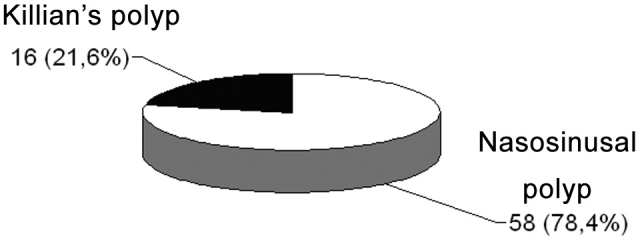
Nasal polyp surgeries

During the analyzed time period, 74 nasosinusal surgeries were carried out in order to treat polyps,16 (21.6%) to treat Killian’s polyps and 48 (78.4%) for nasosinusal polyposis (Chart 6). In this series there were no intra, nor postoperative complication.

## DISCUSSION

Although this is no rare disease, Killian’s Polyps have enjoyed little attention recently, with very few cases described in the specialized literature. It is a benign, unilateral disease, that starts on the floor of the maxillary sinus, near dental roots, and migrates towards the nasal cavity, the cavum, and the oropharynx. Microscopically, it is undistinguishable from the intramural cyst. Syme, and Towbin^11^, found 26 patients (3.0%) with Killian’s polyps in another series with 878 patients with nasal polyps. In Heck and Towbin’s^11^ series, they found 56 patients (3.7%) with antrochoanal polyps among 1,720 patients with nasal polyps. In the large investigation carried out involving 118,374 patients examined in over thirteen years at the Helsinki University Central Hospital, Sirola and Towbin^11^ reported 1,295 cases of nasal polyps, 80 (6.3%) of them were Killian’s Polyps. In this series, they found 74 cases of nasal polyps, and of these, 16 (21.6%) were Killian’s Polyps. This increased incidence in relation to the literature reviewed is linked to the fact that for this study they only considered those patients with histopathologic diagnosis of inflammatory polyps, and did not include other nasal tumors, which would serve as differential diagnosis. Killian’s Polyps happen more commonly in children and young adults. In the present investigation, 11 patients (68.75%) were younger than 21 years. It is considered the most common polyp in children, and its etiology is most likely that of a chronic inflammatory bacterial disease and/oa r cystic fibrosis. Allergy is but a rare cause. Raji et al.8 and Lapantin and Raji et al.^8^ did not show significant differences in relation to its gender-wise incidence. However, Piquet and Raji et al.8 ; and Rugina and Raji et al.8 showed a clear male predominance. In the present study, there is slight male predominance (1:1,3). In their series, Gomes et al.3 presented the major symptoms as being: bilateral nasal obstruction (7/12), sleep snoring (5/12), unilateral nasal obstruction (4/12) and oral breathing (3/12). In their investigation, Raji et al.8 noted the following as main symptoms: unilateral nasal obstruction (9/12), purulent rhinorrhea (8/12), sleep snoring (4/12) and bilateral nasal obstruction (3/12). In the present investigation, unilateral nasal obstruction (13/16) and purulent rhinorrhea (7/16) were the most frequently found symptoms. According to Marshal and Gomes et al.^3^, depending on its size, the antrochoanal polyp may cause Eustachian tube obstruction and thus, unilateral secretory otitis media, which did not happen in the present series. Robson et al.^10^ described the case of a young man with severe epistaxis as the main symptom. In the present study, we found 6 (37.5%) patients with epistaxis, and none of them with severe nasal bleeding. The simple Killian’s Polyp removal may cause recurrence in approximately 25% of the patients^3^. Aktas and Raji et al.^8^ reported 4 cases of recurrence in 8 patients operated by simple resection. It is necessary to identify the polyp origin, because the sinusal component must also be resected, in an attempt to preclude tumor recurrence. Despite rigorously following the advocated surgical techniques, we still had antrochoanal polyp recurrence in two patients (12.5%).

## CONCLUSIONS

1. The antrochoanal polyp happens predominantly in children and young adults.
2. The combined external and endonasal approach was the most used surgical technique.
3. Despite approaching the polyp origin in the maxillary sinus, there is a possibility of postoperative recurrence.
